# Violence against women: prevalence and risk factors in Turkish sample

**DOI:** 10.1186/s12905-017-0454-3

**Published:** 2017-11-03

**Authors:** Selma Sen, Nursen Bolsoy

**Affiliations:** Manisa Celal Bayar University Faculty of Sciences, Midwifery Department, 45100 Manisa, Sehzadeler Turkey

**Keywords:** Domestic violence, Women, Perceptions of domestic violence

## Abstract

**Background:**

This study reports on a large cross-sectional study of violence against women in Turkey, and outlines the risk factors associated with intimate partner violence. The purpose of this study was to identify in order to evaluate the domestic violence against women living in Manisa and to determine the risk factors affecting this situation.

**Methods:**

We implemented a cross-sectional descriptive study in the Manisa province of Turkey. The research data were collected by using a “Women’s Information Form” consisting of 32 items, and “Scale of Domestic Violence Against Women”. The study was conducted with 1760 women who complied with the inclusion criteria.

**Results:**

It was determined that score averages of 30.0% of women from the scale of domestic violence against women were above the score average of the scale (71.38 ± 10.71) and they were exposed to violence more than the others. A statistically significant difference was obtained in the statistical analysis made between score averages from the scale of domestic violence against women and such variables as age, education, employment status, social insurance, immigration status, place of residence, marital age, year of marriage of women; age, education status, employment status of husband; and whether the husband has another wife (*p* < 0.05).

**Conclusions:**

It was also found out that the rate of domestic violence against women is high, women does not perceive many behaviors of their husbands as violence, and the most important factor leading to this situation is social status. It is believed that the results of the study will be a guidance to local authorities, formal and voluntary organizations, educational institutions, and relevant researchers in the prevention of violence against women.

## Background

Violence is an important community health problem that can be encountered in every area of human life and is gradually increasing in the world [1, 2]. Women and children are exposed to the greatest violence in Turkey, as in in all other societies [3].

Violence against women can be defined as all acts of gender-based behavior that is likely to result in psychical, sexual, and psychological harm or suffering to women, or causes to coercion or arbitrary deprivation of liberty, whether occurring in public or in private life [4].

Violence against women are often categorized as emotional, physical, psychological, economic, and sexual violence [5]. Physical violence is a means of intimidation, suppression and sanction of the brute force; sexual violence is the use of sexuality as a way of threat, oppression and control; psychological violence or verbal abuse is the suppression, punishment and control of women with one’s behavior speech; economic violence is defined as the use of economic resources and money as sanction and treat over women [6].

It is notified that 30% of women in the entire world and 37% of women in the East Mediterranean Region, including our country, are exposed to physical and/or sexual violence by their husbands or partners in any period of their lives [2]. The reports of General Principles of European Union agency for fundamental rights (FRA) showed that the amount of spousal abuse in the countries of European Community ranged from 13% to 32% in 2014 [7].

According to the research about violence against women resulted in 2014 in the countries of EU, one of each three women are exposed to physical or sexual violence from the age of 15. Only 14% of the domestic violence cases is reported. One of 10 women above the age of 15 is exposed to sexual violence. Two of five women (43%) remarked that they were exposed to psychological violence by their ex husbands / life partners (25% abasement, 5% being imprisoned in the house etc.) [7].

In Turkey, the government has started to take precautions and important steps for combating violence against women and international developments have accelerated relevant studies. In accordance with international conventions being signed in Turkey, the provisions tolerating discrimination and violence against women in the Civil Code and the Turkish Penal Code have been considerably cleaned up and replaced by provisions aimed at establishing equality [3, 8]. As a recent development, the “Law on the Protection of the Family and Prevention of Violence against Women” was passed on 20 March 2012 and domestic violence against women began to be considered a crime [9].

One of the most discussed diaries in Turkey is violence against women, beyond any doubt. Besides its disgusting appearances with the cases such as murders, violence against women also attracts attention with the rates propounded with the researches. According to the results of Research on domestic violence against women in Turkey, 2009, 39% of women in Turkey are exposed to physical violence, 15% of them are exposed to sexual violence and 44% of them are exposed to emotional violence. According to the results of Research on domestic violence against women in Turkey, 2015, 36% of the women in Turkey are exposed to physical violence. In other words, about 4 of each 10 women are exposed to violence by their husbands or life partners. 12% of the women are exposed to sexual violence and 44% of them are exposed to emotional violence, in the same report [10, 11].

In similar studies conducted in Turkey women’s exposure to violence is detected at the rate of emotional violence is 75%, physical violence is 39%, and sexual violence is 28% [12]. In another study, it was found that psychological violence was at the rate 99.1%, physical violence was 36.4%, and sexual violence was 5.4% [13]. Dindas and Ege reveals that 29% of women are exposed to verbal violence, 25.9% of them are exposed to emotional violence, 14% of them are exposed to physical violence, 11.4% of them are exposed to economic violence, and 8% of them are exposed to sexual violence [14]. In study of Guler and his colleagues [15], it stated that 40.7% of women are exposed to violence [15]. In study conducted by Altınay and Arat [16] on 1800 married women, it was stated that one out of every three women (35%) are subjected to physical violence by their spouse at least once during their lifetime. In Yaman Efe and Ayaz’s study [17], it was found that all of the women in the study are exposed to domestic violence (any type of violence), 54.6% of them were low level, 38.4% were middle level and 7% were exposed to high levels of violence. The result of the researches suggest that the prevalence of women exposed to violence in Turkey and in the World is high [2, 7, 10, 11].

Beside defining the facts of violence against women, treatment, support and rehabilitation, health professionals, especially nurses who are always in contact with the society have important duties in reducing the violence in the society and forming a culture without violence including protection and early intervention. Nurses should do their duties of knowing the victim of domestic violence, encouraging her for expressing her problem without feeling guilty, providing her privacy and security, collecting appropriate data, directing her to other professionals when needed and guiding about support systems [18]. Because nurses have duties in a number of different fields, they have a considerable impact to prevent, treat, and reduce the domestic violence. Nurses have the opportunity to observe, influence, and train the families in where they are. For these reasons, nurses could have multiple help and contributions to the victims of violence [19]. In addition, nurses should have knowledge of the households and other environments where violence is experienced, should break the cycle of violence, and ensure to reduce of the long or short term effects of violence on the women and her family [20]. It is thought that nurses being involved in advocacy and counseling roles is essential for strategies to prevent violence against women, so women who are exposed to violence could be informed about their legal rights and guided to the appropriate channels.

In recent years, different studies conducted in Turkey showed that one out of every three women is exposed to violence at some point in their lives. Even though the lack of statutory data on violence against women is causing to have limited information whether violence against women increased or not, the high level of figures suggests that it is a multidimensional social problem which is fueled by structural dynamics. For this reason, it necessary in the case of Turkey to understand the different dimensions of violence thoroughly and analyzes their different aspects. In this sense, understanding the dimensions of violence against women and specific to Turkey, analyzing the aspects of violence against women based on gender inequality are needed. Due to these reasons, this study was conducted in order to evaluate the domestic violence against women living in Manisa and to determine the risk factors affecting this situation.

## Methods

### Participants and design

The study was conducted in the central district of Manisa between 15 November 2013 and 01 January 2014. The province of Manisa where the research is conducted is in the West of Turkey, in the Aegean Region. 50.1% of the population are men and 49.9% of the population are women. Yearly population growth rate is 9.83%. The amplitude of the young population in the province of Manisa attracts attention. 36% of the population of the province is under 25 according to 2013 census. When the data of the province of Manisa about education is evaluated, two aspects attract attention: the data of the province is above the average of Turkey in terms of literacy and schooling and there are still problems about the inadequacy of the level of education. The rate of illiterate people over the age of 15 throughout the province of Manisa is 3.6%. 83% of the illiterate people are women and 17% of them are men [21].

The Scale of Domestic Violence Against Women which we used in our research is applied to women who have married once or have lived together with a partner. Because of the fact that the majority of women and men in our country prefer official marriage, married women were included in the study. The target population of the study consisted of women aged 18–88 living in 13 towns and 85 villages in Central Manisa (According to the 2012 Turkish Statistical Institute Address-Based Population Registration System, in the central district of Manisa *N* = 677.366). The minimum sample size of the study, which was exemplify of universe which was calculated as 1038 people by using the Epi info 7.0 software and taking the frequency of domestic violence against women in our society as 42%, confidence limit 95% and the margin of error 3%. As there were likely to be losses in the sample, the sample size was determined as 2000 women. Women over 18 who married at least once were included within the scope of the research. Women to be included in the study were selected from the registration of the Department of Data Processing, Unit of Support Services, Manisa Provincial Public Health Directorate via stratified (according to urban, semi-urban and rural area rates), simple random sampling. People chosen and determined randomly from the family serial numbers from the registries of cities, half-cities and rural areas were included in the sample.

### Questionnaires

In the study, the data were collected by using the “Women’s Information Form” consisting of 32 items which was prepared by the researchers in accordance with literature and the “Scale of Domestic Violence Against Women”. All data collection tools were used in Turkish, in a way the participans could undertand.

The women’s information forms consisted of questions about their socio-demographic and marital features, income status, residence, family type (nuclear, extended etc.) and educational background.

### Scale of domestic violence against women

Developed by Kılıc in 1999, Scale of Domestic Violence against Women determines domestic violence committed by the husband on the woman. The scale consists of 50 items and 5 sub-dimensions. Sub-dimensions are physical violence, emotional violence, verbal violence, economic violence and sexual violence. Each group can be used independently. Each sub-dimension consists of 10 items. Items related to physical violence are numbered 1, 6, 11, 16, 21, 26, 31, 36, 41, 46 while emotional violence are expressed in the items numbered 2, 7, 12, 17, 22, 27, 32, 37, 42, 47. Sub-dimension concerning verbal violence includes the items numbered 3, 8, 13, 18, 23, 28, 33, 38, 43, and 48 while sub-dimension concerning economic violence includes the items numbered 4, 9, 14, 19, 24, 29, 34, 39, 44, and 49. The sexual violence related items are numbered 5, 10, 15, 20, 25, 30, 35, 40, 45, and 50. The total score obtained from the scale shows the level of “domestic violence against women”. The scale is a likert type scale from 1 to 3 with responses of “Never”, “Sometimes” and “Always”. Participants obtained scores from each statement in the scale as follows: Never (1), Sometimes (2), Always (3). Out of 50 items, 16 items numbered 2, 5, 7, 8, 9, 12, 14, 22, 28, 30, 32, 33, 38, 44, 47 and 49 were reversely coded. The lowest score to be obtained from the scale is 50 while the highest score to be obtained from the scale is 150. The lowest and highest scores to be obtained from each sub-dimension are 10 and 30, respectively. High scores that women get from the scale show high level of exposure to violence while low scores indicate low level of exposure to violence. Cronbach alpha coefficients of the scale and sub-dimensions were determined to range between 0.73 and 0.94 [22]. In this study, Cronbach alpha coefficient of the scale was calculated as 0.71.

### Procedure

In the first stage of the study, an announcement was made to midwifery and nursing students and students who applied to work as poll takers in the project were selected (40 midwifery and 10 nursing students). Poll takers were trained by researchers for 1 day in the subject, the content of the study, ethical issues and how to apply the forms to be used. After completing training, poll takers were separated into groups of 10 individuals and each group was put under the responsibility of one researcher. The required permissions were obtained from the governorship for using the questionnaires and the relevant district managers (mayors and local leaders) were informed accordingly.

In the second stage of the study, the poll takers went to the addresses specified in the sample and informed individuals about the study and they collected data from the women who volunteered to women in the study. The data of the research was collected by pollsters door-to-door, in the houses of the people determined with the face to face meeting method. The questions of the survey were read by the pollsters and the polls were completed in accordance with the discourse of the participants. The addresses of women who refused to participate in the study or were not available were noted and a total of 2000 women were interviewed. The Women’s Information Form and Scale of Domestic Violence Against Women were used for the 2000 female who were interviewed. The transportation and lunch expenses of poll takers were paid within the scope of the project.

In the third stage of the study, the researcher responsible for each group collected the data, checked them and recorded them on the database. Incomplete and erroneous forms were excluded and the data from a total of 1760 women were recorded on the system.

### Statistical analysis

Descriptive data are presented as number, percentage and mean. The data gathered from the groups were compared with the Paired Sample T Test, Mann whitney U test, One-Way ANOVA for Repeated Measures Test. All analyses were carried out using the SPSS for Windows, release 15 .0 (SPSS, Inc., Chicago, IL, USA). A *p* value of <0.05 was thought to be crucial for all analyses.

## Results

Considering the descriptive features of women comprising the study group, it was determined that the women had an age average of 37.15 ± 12.14. Considering their educational background, it was determined that 5.9% of the women were illiterate, 6.8% had never gone to school but they were literate, 12.1% were university graduates. It was also determined that 27.5% of the women worked, that the women had a lower educational level and their rates of employment were very low in general (Table 1).

**Table 1:** Some Desciriptive Characteristics of The Womens, Manisa 2014

Characteristics		Women	
		n	%
Age		X ± SD (37.15 ± 12.14) Min:18 Max:88	
	18–27 age	421	23.9
	28–37 age	579	32.9
	38–47 age	399	22.7
	48–57 age	248	14.1
	58–67 age	82	4.6
	68 age and ↑	31	1.8
Education Status	İlliterate	104	5.9
	Literate	120	6.8
	Elemantary School	605	34.4
	Secondary School	320	18.2
	High School	398	22.6
	College	213	12.1
Employment Status	Working	484	27.5
	Unemployed	1276	72.5
Socioeconomic Status (n= 1756)	Low	227	12.9
	Middle	1366	77.6
	Well	163	9.5
TOTAL		1760	100.0

With regard to women’ exposure to violence in individuals in the study group, it was determined that women were mostly exposed to verbal (61.8%) and physical violence (54.8%). Women were exposed to violence mostly from their husbands and fathers. According to the declarations and perceptions of the participants in our study, 16.2% of the women stated that they frequently experienced violence. It was determined that women exposed to violence usually preferred to keep silent (Table 2).

**Table 2:** State of Being Exposed to Violence in Women Comprising the Study Group, 2014

Feature		Women	
		n	%
*State of being exposed to domestic violence	Yes	512	29.1
	No	1248	70.9
**Types of Violence Women (*n* = 512) Men (*n* = 471)			
Physical violence	Yes	272	54.8
	No	224	45.2
Emotional violence	Yes	218	44.0
	No	278	56.0
Sexual violence	Yes	21	4.2
	No	475	95.8
Verbal violence	Yes	307	61.8
	No	189	39.2
Economic violence	Yes	36	7.4
	No	460	92.6
Person committing violence Women (n = 488)*** Men (n = 400)***	Mother	38	7.8
	Father	82	16.8
	Sibling	7	1.4
	Partner	343	70.3
	Mother-in-law	16	3.3
	Father-in-law	2	0.4
Frequency of being exposed to violence Women (n = 496) Men (n = 437)	Rarely	154	31.0
	Sometimes	155	31.3
	Generally	67	13.5
	Frequently	95	19.2
	Always	25	5.0
Behavior being displayed when exposed to violence Women (n = 507) Men (n = 467)	I kept silent	334	65.9
	She/he said she/he was sorry and I was reconciled	115	22.7
	I went to the police station	8	1.6
	I left home	21	4.1
	Other	29	5.7
TOTAL		1760	100.0

*The data that were acquired in the table show the verbal statements of individuals and no scale was used

**Among the types of violence being committed, more than one options were marked

***The first person committing the violence was taken into consideration

When the distributions of the score averages women received from the scale of domestic violence against women are examined, it is seen that 30.0% of women have score averages above the score average of the scale (71.38 ± 10.71) and they are exposed to violence more than the others (Table 3).

**Table 3:** Distribution of Average Score of Scale for Domestic Violence against Women

Scale for Domestic Violence against Women (n = 1760)	Number	Percent	X ± SD
Total score average			
Below the average scale score 50–74	1181	70.0	71.38 ± 10.71 (Min:54.00 Max: 127.00)
Above the average scale score 75–150	508	30.0	
Subscales			
Physical violence			
Below the average scale score (15↓)	1643	94.6	10.80 ± 1.96
Above the average scale score (15↑)	92	5.4	
Emotional violence			
Below the average scale score (15↓)	386	22.2	16.93 ± 2.68
Above the average scale score (15↑)	1358	77.8	
Verbal violence			
Below the average scale score (15↓)	844	48.9	15.16 ± 3.06
Above the average scale score (15↑)	884	51.1	
Economic violence			
Below the average scale score (15↓)	587	33.8	15.97 ± 2.69
Above the average scale score (15↑)	1152	66.2	
Sexual violence			
Below the average scale score (15↓)	1463	84.1	12.54 ± 2.72
Above the average scale score (15↑)	276	15.9	

When the sub-dimension score averages of the women are examined, it is determined that physical violence sub-dimension score averages of 5.4% of the women are above the average of the sub-dimension (10.80 ± 1.96) and they are exposed to physical violence more than the others; emotional violence sub-dimension score averages of 77.8% of the women are above the average of the sub-dimension (16.93 ± 2.68) and they are exposed to emotional violence more than the others; verbal violence sub-dimension score averages of 51.1% of the women are above the average of the sub-dimension (15.16 ± 3.06) and they are exposed to verbal violence more than the others; economic violence sub-dimension score averages of 66.2% of the women are above the average of the sub-dimension (15.97 ± 2.69) and they are exposed to economic violence more than the others; and sexual violence sub-dimension score averages of 15.9% of the women are above the average of the sub-dimension (13.63 ± 2.77) and they are exposed to sexual violence more than the others (Table 3).

A statistically significant difference was obtained in the statistical analysis made between the score averages of the women from the scale of domestic violence against women and such variables as age group, education status, employment status, social insurance status, immigration status, place of residence, marital age, year of marriage of woman; age, education status and employment status of the husband; and whether the husband has another wife (*p* < 0.05) (Table 4).

**Table 4:** Comparison of Some Desciriptive Characteristics of The Womens According to Scale for Domestic Violence against Women Averages

Characteristics (n = 1760)		X ± SD	t	p ^a^
Age	Below average (952)	69.89 ± 11.30	−2.05	0.04
	Above average (737)	71.04 ± 11.66		
Education Status	< Primary and Primary school (799)	72.53 ± 11.94	7.36	0.00
	≥Secondary school (890)	68.47 ± 10.68		
Employment Status	Working (463)	68.25 ± 11.85	−4.75	0.00
	Unemployed (1226)	71.20 ± 11.24		
Social Assurance	Yes (1620)	70.17 ± 11.24	3.88	0.00
	No (69)	75.63 ± 15.06		
Duration of living in Manisa	Since she was born (1060)	69.50 ± 10.61	−4.18	0.00
	Came with migration (629)	71.90 ± 12.66		
Place of Residence ^b^	City (1207)	70.12 ± 11.44	16.51	0.00
	Rural area (203)	67.39 ± 10.40		
	Squatter (350)	73.08 ± 11.63		
Marital Age (21.10 ± 3.64)	Below average (1031)	72.05 ± 11.72	7.55	0.00
	Above average (658)	67.80 ± 10.56		
Year of Marriage (15.55 ± 12.27)	Below average (923)	69.22 ± 10.79	−3.74	0.00
	Ortalamanın üstü (721)	71.31 ± 11.72		
Husband Age (40.29 ± 12.23)	Below average (950)	69.60 ± 11.09	−2.26	0.02
	Above average (694)	70.88 ± 11.44		
Education Status of The Husband	< Primary and Primary school (614)	72.20 ± 12.01	4.91	0.00
	≥Secondary school (1075)	69.36 ± 11.02		
Employment Status of The Husband	Working (1574)	69.95 ± 11.06	−2.29	0.02
	Unemployed (138)	72.28 ± 13.11		
whether the husband has another wife ^c^	Yes (24)	78.42 ± 15.67	4.66	0.00
	No (1688)	70.08 ± 11.16		
Women Experience Violence Situations	Yes (512)	78.82 ± 13.33	21.92	0.00
	No (1248)	66.93 ± 8.46		

^a^The independent-samples t-test *p*-value

^b^One Way ANOVA test f and p-value

^c^Mann whitney U test

According to the findings, women who are aged above the age average (37.15 ± 12.14); have education levels equivalent to and lower than primary education; have no social insurance, have come to the region through immigration; live in squatter settlements; have below-average marriage ages (21.10 ± 3.64); have above-average year of marriage (15.55 ± 12.27) have higher total violence scores and are exposed to violence more than the others. Also, the women whose husbands are aged above the average (40.29 ± 12.23); have education levels equivalent to and lower than primary education; have unemployed wives; and have other wives are reported to have higher total violence scores and be exposed to violence more than the others. Lastly, women who expressed to have suffered from domestic violence (78.82 ± 13.33) have higher total violence scores and are exposed to violence more than the others. However, it is seen that women who expressed not to have suffered from domestic violence (66.93 ± 8.46) have considerably high score averages in the scale of domestic violence against women.

## Discussion

Violence against women is a social problem that, at the most basic level, threatens women’s lives and which prevents them from participating fully in social and cultural life. It remains current in Turkey, as in many other countries.

In Turkey the proportion of married women who stated that they had been exposed to physical violence is 36%. In other words, approximately 4 out of every 10 women had experienced physical violence from their husbands or partners [10]. In another study of women, 40.7% of women stated that they had been exposed to domestic violence. Among these women, 91% had experienced violence from their husbands, 22.7% from their husbands’ relatives and 19.7% from their own relatives [15]. In our study, it is determined that women are exposed to physical violence most and this violence is practiced by their husbands firstly and by their fathers (men) secondly. In the most of the studies carried out in Turkey, exposure to violence rates of women vary between 40% and 80%. [10, 11, 17, 23, 24]. Our findings show a parallelism with the findings across the world. It is possible to suggest that women mainly experience violence from men.

It has been observed that women are exposed to violence by their husbands no matter what educational level, social status or economic conditions they or where they live [3]. It has been noted that 30% of women in the world and 37% of women in the eastern Mediterranean region, including in Turkey, are exposed to physical and/or sexual violence by their husbands or partners at some time in their lives [2]. The reports on General Principles of European Union Agency for Fundamental Rights (FRA) showed that the amount of spousal abuse in the countries of European Union ranged from 13% to 32% in 2014 [7].

Although most of the women are exposed to domestic violence, studies conducted show that some women are exposed to violence more than the others [25–27]. The basic factors that determine the social status of women involve education, employment and social security. The right to education and employment is among the rights of women on the basis of fundamental human rights. However, it is known that women’s participation in education and business life is obstructed by families, relatives and acquaintances across the country [10]. One of the most important risk factors for violence against women is the lower educational level of women [26, 27]. As the educational level of women decreases, the rates of being exposed to violence increase. While one out of every five women with a higher education is exposed to violence, one out of every two women with a lesser degree of education are exposed to violence [3, 10]. Increasing women’s educational levels in general will develop their personal skills, employability and consequently their socio-economic levels. It will thus decrease their risk of being exposed to violence [1, 28]. In our study, it was found out that women with education levels equivalent to and lower than primary education have higher total violence scores and are exposed to violence more than the others.

In Turkey, the educational level of women is not at the required standard. According to the National Research into Violence against Women that was conducted in Turkey 2014, 19% of men and 32% of women are either illiterate or, despite being literate, have never gone to school, while only 10.1% of men and 6.1% of women are university graduates [10]. According to the Population and Health Research that was conducted in Turkey in 2013, 16.1% of men and 28.2% of women are either illiterate or, although literate, have never gone to school, while 29.1% of men and 20.5% of women completed high school and higher education [21]. In both studies, the rate of illiteracy or of lack of attendance at school despite being literate was almost twice as large among women than men. In our study, there is a 50% difference between the rates of illiteracy/literacy without schooling among women and men to be illiterate/only literate and to be university graduates. The study results show a parallelism with studies being conducted country-wide in which women have lower educational levels than men. Considering the employment rates of women and men, it can be observed that almost three women out of four are unemployed and have no independent income which is noteable. Women and men have similar average ages and it is possible to assert that women have a lower social status in terms of their educational level and working conditions compared to men.

In a study conducted in Pakistan, it was determined that women getting married at young ages are exposed to domestic violence more than the women getting married in adulthood and are deprived of education and social insurance due to early marriage [25]. “Research on Domestic Violence against Women in Turkey (2009)” which covers the whole country reports that physical and sexual violence increases with the advancing age (45–59 age group, 47.9%), but when the last 12 months are taken into consideration, young women (15–24 age group, 21.0%) are exposed to violence more than the other age groups. Researchers concluded that women suffer from violence at younger ages and the first years of the marriage [11]. In our study, total violence scores of the women who have above-average ages and years of marriage and get married at earlier ages were found to be higher. It was concluded that as age and years of marriage increase, the rate of lifetime exposure to violence increases and women who get married at young ages are exposed to violence more than the others.

According to another study conducted throughout Turkey, the percentage of men who commit physical violence among those aged 45 and over (36.8%–42.5%) is higher than the rate of men who commit violence in 15–24 age group (19.7%). As the age of men in a group increases, the rate of those who commit physical violence increases [10]. Our study shows that total violence scores of women whose husbands are aged above the average (40.29 ± 12.23) are higher and they are exposed to violence more than the others. It is thought that the rate of committing lifetime violence increases among men as their ages and durations of marriage increase.

The studies conducted so far reported certain major factors affecting women’s exposure to violence, which include low education level of the husband, unemployment of the husband and the fact that the husband has another wife [10, 11, 17, 23, 24, 29, 30]. In parallel to these studies, our study revealed that women with husbands who have education levels equivalent to and lower than primary education, who are unemployed, and who have more than one wives are exposed to violence more than the others.

In the literature, it is noticeable that women suffering from domestic violence have low education and socio-economic levels and do not work in any income-generating jobs [10, 11, 17, 23, 24, 29–31].

In our study, the relation between violence and a woman who is not working and lacks social insurance displays a similar pattern to the relation between education level and violence. When it is thought that education level, employment status and social insurance play key roles in terms of the welfare levels of the women, it can be stated that women with lower welfare levels are exposed to violence more than the others. Similarly, the study conducted throughout Turkey shows that one of two women who have low welfare levels (49.9%) and one fourth of all women with high welfare levels (28.7%) are exposed to violence in any period of their lives [11]. Both literature and results of this study have demonstrated that exposure to violence increases among women as their levels of welfare decrease.

Previous studies conducted in Turkey reported that the frequency of domestic violence against women differs depending on the geographical regions where women live [10, 11, 17, 23, 24, 30, 31]. It was determined in our study that women who migrated to the region in question from the eastern part of Turkey and live in shatter settlement have higher total violence scores and suffer from violence more than the other women. Although the region where the study was conducted is located in the western part of Turkey, the results of the present study share similarity with the other studies conducted throughout the country since most of the women living in shatter settlement migrated to this region from eastern Turkey [10, 11, 17, 23, 24, 31].

It is seen in our study that scale score averages of the women who state that they do not suffer from violence are outstandingly high (66.93 ± 8.46). It can be understood from our study that a considerable number of women do not perceive many behaviors of their husbands as violence and their awareness concerning violence are inadequate. This finding may imply that women do not perceive certain violent behaviors of their husbands as violence. Similar results were obtained in previous studies [11, 15, 32]. As a result of the influence of the cultural values in the patriarchal social structure in Turkey on women, they are convinced that their husbands have the right to beat them. These women think that it is normal to be exposed to violence as other women also experience violence from their husbands [32].

### Implications

It was determined that domestic violence against women is still common throughout Turkey and women do not perceive many behaviors of their husbands as violence, and the main factors contributing to this situation are social status and level of welfare. When the findings of the present study are evaluated in combination with the results of the previous studies, it becomes evident that they are similar to a large extent.

### Limitations

Our study has several limitations. Although the first intention was to conduct the study throughout Turkey, it was carried out in a specific region due to financial difficulty and time constraints. During the study, data were collected via personal statements. Despite the similarities between the findings of the present study and results of the studies covering the whole country, the results of this study only belong to the region where it is carried out and cannot be generalized to Turkey. Finally, the cross-sectional and descriptive design of the study limits conclusions about causality for some findings.

## Conclusions

As a conclusion, as in many studies on violence, this study reports that the rate of domestic violence against women is high, women do not perceive many behaviors of their husbands as violence, and the main factor leading to this situation is social status. Major factors increasing domestic violence are reported to include relatively advanced ages of women and their husbands, low education levels, marriage at young ages, unemployment, lack of social insurance, the fact that husband has another wife, immigration and living in shatter settlement.

Domestic violence against women is preventable. Approach of the society is of great importance in preventing violence and abuse. Societies should take action against violence cases through government programs, legal arrangements, media, official and voluntary organizations, education institutions etc. in order to prevent violence. It is believed that the results of the study will be a guidance to local authorities, formal and voluntary organizations, educational institutions, and relevant researchers in the prevention of violence against women.
